# Modelling cerebellar Purkinje cells with simple neuron models of the threshold type

**DOI:** 10.1186/1471-2202-14-S1-P174

**Published:** 2013-07-08

**Authors:** Eoin P Lynch, Conor J Houghton

**Affiliations:** 1 School of Mathematics, Trinity College Dublin, Dublin, Ireland; 2 Department of Computer Science, University of Bristol, Bristol, UK

## 

The Purkinje cells of the cerebellum are responsible for processing the inputs necessary for coordinating movement.

In the cerebellum, cells receive external input from only two sources, climbing fibres and mossy fibres [1]. Mossy fibres excite granule cells whose axons, called parallel fibres, enervate Purkinje cells. The integration of inputs from tens of thousands of individual parallel fibres synaptically connected to a single Purkinje cell causes it to emit a 'simple spike' discharge. This is a single spike with the waveform of a typical action potential.

In addition to simple spikes, cerebellar Purkinje cells can produce a second kind of electro-physiological response called a complex spike only after activation by climbing fibre inputs. The complex spike consists of an initial spike followed by sub-threshold oscillations in the membrane potential which last for tens of milliseconds. This dual responsiveness of Purkinje cells presents a challenging modelling problem. Modelling efforts have usually relied on complicated conductance based models with many compartments.

On the other hand, recent results [2,3] have shown that in the case of regular cortical neurons, certain simple neuron models which feature adaptation are capable of reproducing the behaviour of real cortical neurons and of detailed Hodgkin-Huxley type models of the same to a high degree of accuracy. Two notable examples of these recent models are the adaptive exponential integrate and fire model of Brette and Gerstner [2] and the multiple adaptation time-scales (MAT) model of Kobayashi et al. [3] which is a simple integrate and fire equipped with multiple scales of adaptation in the voltage threshold.

Here we evaluate how effectively the rich dynamics of cerebellar Purkinje cells can be modelled using specially designed modifications of simple neuron models of this type. We use recently developed neuron model optimisation tools for this purpose [4] and benchmark the model performance against detailed multi-compartmental conductance based models in NEURON and Genesis[5].
